# Differences in infant feeding practices between Chinese-born and Australian-born mothers living in Australia: a cross-sectional study

**DOI:** 10.1186/s12887-018-1157-0

**Published:** 2018-06-28

**Authors:** Kristy A. Bolton, Peter Kremer, Kylie D. Hesketh, Rachel Laws, Konsita Kuswara, Karen J. Campbell

**Affiliations:** 1 Institute for Physical Activity and Nutrition (IPAN), School Exercise and Nutrition Sciences, Deakin University, Geelong, Australia; 2 Centre for Sport Research, School of Exercise and Nutrition Sciences, Deakin University, Geelong, Australia; 3 Centre for Research Excellence in the Early Prevention of Obesity in Childhood, Sydney, Australia; 4 Centre for Obesity Prevention and Management Research Excellence in Primary Heath Care (COMPaRE-PHC), Sydney, Australia

**Keywords:** Early childhood, Breastfeeding, Ethnicity, Immigrants, Culture, Overweight, Obesity, Maternal child health

## Abstract

**Background:**

Chinese immigrants are the third largest immigrant group in Australia. Recent qualitative evidence from Victorian Maternal and Child Health nurses indicate that infants of Chinese parents commonly have rapid growth trajectories and that high value is placed on rapid growth and having a fatter child; with low breastfeeding rates and overfeeding of infant formula. The aim of this study was to compare infant feeding practices (breastfeeding, infant formula, other liquids, solids) of Chinese-born and Australian-born mothers living in Australia.

**Methods:**

Using the Australian National Infant Feeding Survey dataset (2010–2011), infant feeding data from Chinese-born mothers (*n* = 602) were compared with a random sub-sample of Australian-born mothers (*n* = 602). Group differences on feeding practices were tested using Chi-square or t-tests and the effect of ethnicity on infant feeding behaviours assessed using regression.

**Results:**

Compared to infants of Australian-born mothers, infants of Chinese-born mothers were younger when they first consumed infant formula, water-based drinks and fruit juice and older when they first ate solid foods (*p* < 0.05). Furthermore, infants of Chinese-born mothers were less likely to have ever had cow’s milk (OR: 0.37 95%CI:, 0.18–0.78) and solids (0.41, 0.25–0.68); but were more likely to have ever had infant formula (2.19, 1.32–3.62), water (2.45, 1.55–3.87), toddler milk (3.39, 1.60–7.18), water-based drinks (e.g. cordial, soft drink, tea; 2.48, 1.12–5.49), and fruit juice (4.03, 2.50–6.51). Those ≤4 months of age were more likely to have had water-based drinks (7.77, 1.96–30.77) and fruit juice (3.44, 1.14–10.38) (*p* < 0.05) compared to infants of Australian-born mothers.

**Conclusion:**

Differences in mothers’ early infant feeding practices exist between Chinese-born and Australian-born mothers living in Australia. Better understanding these ethnically patterned infant feeding practices is important for identifying key opportunities to promote best nutrition and growth in early life in different ethnic groups within our population.

## Background

Australia is a multicultural country with a population of almost 25 million [1]; and a large immigrant population (28.2%) [2]. In 2016, Chinese were the third largest immigrant group in Australia and represented 2.2% of the total Australian population [2]. The prevalence of overweight and obesity in Australian children is high (27.4% of children aged 5–17 years were overweight or obese) [3], however little is known about the prevalence or predictors in ethnic subgroups. In Australia, ethnic backgrounds (such as Asian, North African; Middle Eastern; Southern, South Eastern and Eastern European) have been demonstrated to increase the risk of overweight and obesity and adverse weight-related behaviours (e.g. physical activity, fitness, diet) in Australian primary-school aged children [4, 5]. In a sample of Australian preschoolers (4–5 year olds), 15.2% were reported to be overweight and 5.5% obese; however in those who spoke a language other than English at home, the prevalence was higher with 16.9% overweight and 8.4% obese [6]. Evidence about the prevalence of overweight and obesity in children of Chinese-born immigrants is scarce. One study reports primary school-aged low socioeconomic Asian boys to be more likely to be overweight/obese compared to their English-speaking counterparts [4]. It is important to expand these findings and investigate obesity promoting behaviours in immigrant populations to identify opportunities to establish health promoting behaviours early in life.

Disparities in weight status between ethnic and racial groups are thought to be influenced by complex interactions between genetics, physiology, culture, socioeconomic status, and the environment [7]. Cultural factors including language, religions, health beliefs, values, and behaviours may influence the propensity for child and adult obesity [5]. An example is the common cultural misconception that a fat baby is a healthy baby [8]. Additionally, grandparent attitudes, indulging behaviours and poor health knowledge when caring for their grandchildren may contribute to risk of childhood obesity [9]. Our own qualitative work with Chinese-immigrant mothers in Australia has highlighted that Chinese grandparents give mothers advice that goes against dietary guidelines. For example, grandparents may advise mothers to give water, honey and rice porridge water to their infants prior to 4 months of age for extra nutrients. They also promote the use of formula at night so that the mother can rest as this is believed to promote higher breast milk production [10].

Building upon our qualitative work with Chinese-immigrant mothers (hereon named Chinese-born mothers), evidence from qualitative work with key providers of universal infant healthcare in Victoria, Australia (Maternal and Child Health (MCH) nurses), from an area with a high proportion of Chinese-born immigrants indicate rapid growth trajectories to be common in the infants of Chinese-born immigrant mothers (unpublished data). These reports are of concern given that rapid growth in infancy has been demonstrated to be a risk factor for childhood obesity [11–14]. Other reported predictors of rapid growth include early childhood feeding practices such as supplementation or substitution of breast milk with formula and early introduction of solids [15–19]. This immigrant group is reported to highly value rapid growth and a fatter child (unpublished data); have lower exclusive breastfeeding rates [20] and early introduction of infant formula [21]. Further evidence from a recent qualitative study with Chinese-born immigrant mothers in Victoria, Australia shows that conflicting views about infant feeding from grandparents reduced mothers’ confidence to breastfeed exclusively [10].

Early feeding practices and maternal factors have been associated with rapid weight gain in infancy and later obesity. The current Australian Dietary Guidelines recommend infants be exclusively breastfed until around 6 months of age, when solid foods are introduced, and continued until 12 months of age or as long as the mother and child desire [22]. Evidence for breastfeeding being a risk factor for overweight and obesity is contradictory [23, 24]. However, overall it appears that breastfeeding is protective against rapid growth [25] and childhood obesity [15, 16, 26]. In contrast, formula feeding may contribute to the risk for overweight and obesity [27, 28]. Potential mechanisms include the different macronutrient composition of formula; hormonal and microflora components which may influence infant growth rates due to absorption and storage of energy; and poor appetite self-regulation due to maternally controlled feeding practices [29]. Introducing complementary foods very early (i.e. before 4 months of age) may also increase the child’s risk of being overweight [17, 30], however inconsistencies in adjusting for confounding variables limits conclusions. Other maternal predictors of infant rapid weight gain include high pre-pregnancy body mass index (BMI), excess gestational weight gain and smoking whilst pregnant [11].

Data on the rates of breastfeeding, formula feeding and timing of introducing solids to infants of Chinese-born immigrants living in Australia is scarce. Therefore, the aim of this study was to compare the infant feeding practices such as breastfeeding, infant formula; and the timing of introduction and exposure to other liquids and solids of Chinese-born to Australian-born mothers living in Australia. This information may reveal intervention opportunities to promote healthy infant feeding practices and nutrition in this immigrant population and reduce their higher risk of overweight and obesity later in life.

## Methods

### Study design and participants

In 2010–2011 The Australian Institute of Health and Welfare (AIHW) conducted The Australian National Infant Feeding Survey (NIFS) [31], a large scale, national survey of infant feeding practices and behaviours of infants aged 0–24 months. Access to the data source in this study was approved by the data custodian Australian Data Archive.

Children aged up to 24 months were randomly selected nationwide from the universal Medicare enrolment database [31]. The sampling methodology has been previously described [31], but of note is the oversampling of infants aged up to 6 months in order to obtain quality estimates of breastfeeding intensity and duration for this age period and allow comparisons with future national survey data [31]. A primary approach letter was sent to the primary card holder on the child’s Medicare card inviting them to participate in the study [31]. A survey questionnaire was sent to participants with a reply paid envelope and an option of completing the survey online [31]. The final sample size was 28,759 (response rate = 56%) [31]. The current study utilises data from two groups within the full NIFS dataset – all mothers born in China (*n* = 602) and a randomly selected sub-sample of mothers born in Australia (*n* = 602). This random sub-sample was selected using a command in the statistical package Stata which randomly extracted *n* = 602 mothers born in Australia from the total sample (SE 15; StataCorp, College Station, TX, USA).

### Measures

A survey was developed by Australian Bureau of Statistics (ABS) for the AIHW to capture the prevalence and duration of breastfeeding, feeding practices, and the barriers to initiating and continuing breastfeeding as reported by mothers/carers of infants [31]. A literature review informed survey question design and was piloted with parents of 1000 randomly selected children drawn from the Medicare Australia enrolment database. The final survey consisted of 101 questions, 39 of which were utilised for this study.

### Sample characteristics

Demographic information collected included mother/infant date of birth, mother/infant country of birth, postcode, the main language spoken at home, questions regarding a spouse/de facto partner, schooling and educational qualifications, employment, total gross household income, medical issues of the mother (e.g. mental health) [31]. Deciles and quintiles describing area level socioeconomic status were calculated by the custodian of the data using Socio-Economic Indexes for Areas (SEIFA) score of relative disadvantage [31]. Ethnicity of mothers was determined by reported country of birth (i.e. China or Australia). Chinese-born mothers were defined as being born in China (excluding Special Administration Regions such as Hong Kong, Macau, Wolong; and Taiwan Province) [32] and now living in Australia. Regarding Australian-born mothers (non-immigrants); participants were excluded from the random selection if the main language spoken at home was not English (*n* = 6). Mothers reported the recorded birth weight and length of the infant, and self-reported weight and height at the start of their pregnancy and at the date of survey completion [31].

### Infant feeding practices

Mothers reported on infant feeding practices (types of liquid and solid consumption), and the ages when these practices first occurred (months). Mothers reported current breastfeeding (yes/no); whether the infant had ever had formula, water, cow’s milk, toddler milk, soy milk, water-based drinks, fruit juice (yes/no for all); and the age that these items were introduced (number of months recorded in an open field, or less than 1 month). *Water* included any sips of water, and excluded water combined with other liquids (e.g. cordial) or solids (e.g. formula). *Cow’s milk* and *soy milk* included any sips of these milks, including flavoured and powdered milks but excluded these milks combined with solid foods (e.g. cereal). *Water-based drinks* included cordial, soft drink, tea and excluded diluted fruit juice, and infant formula products. *Soft, semi-solid and solid foods* included custards, mashed food diluted with water, milk or other fluids [31].

### Statistical analysis

Descriptive statistics (means and standard deviations (SD), or proportions) were used to summarise data for Chinese-born and Australian-born mothers and their infants. Differences between ethnic group (Chinese- versus Australian-born) and infant feeding practices were tested using Chi-square tests or t-tests. Multiple linear regression examined the influence of ethnicity on the age these items were first introduced. To examine early introduction of water-based drinks, fruit juice and solids, age was dichotomised into ≤4 months, and > 4 months. Binary logistic regressions were conducted to examine the influence of ethnicity on dichotomous variables (yes/no) including infant feeding practices (ever breastmilk, cow, toddler and soy milk, formula, water, water-based drinks, fruit juice, solids) and dichotomous age of introduction (≤4 months, and > 4 months). Analyses were conducted on each variable as unadjusted (model 1), and fully adjusted (model 2) using the following apriori covariates: maternal age, area level of disadvantage (SEIFA), infant age at the time of survey completion, presence of a spouse pre- and post- birth, educational qualifications, income, parity, current smoking status and pre-pregnancy BMI. In all cases effects were considered statistically significant at *p* < 0.05.

### Ethical approval

Approval for this study was originally provided by the Australian Institute of Health and Welfare’s Ethics Committee. The secondary data analysis conducted in the current project was approved by the Deakin University Human Research Ethics Committee (2014–161).

## Results

There were differences between Chinese-born and Australian-born mothers on several demographic variables (Table 1). Compared to Australian-born mothers, a higher proportion of Chinese-born mothers were older; experienced less socioeconomic disadvantage; had a spouse at the time of birth; were more educated and had higher level qualifications; and were primiparous. Mean BMI was lower in Chinese-born mothers and consequently a higher proportion were classified as underweight and healthy weight at the start of the pregnancy and upon survey completion post pregnancy. A higher proportion of Australian-born mothers had higher gross total income; had three or more children; and were more likely both to have smoked whilst pregnant and to currently smoke. Regarding main language spoken at home, 15% of Chinese-born mothers spoke English. Infants of Australian-born mothers were significantly heavier at birth compared to infants of Chinese-born mothers (mean ± SD; 3.43 ± 0.63 kg vs. 3.34 ± 0.68 kg, respectively, *p* < 0.05).

**Table 1:** Demographic characteristics of Chinese-born and Australian-born mothers living in Australia

	Chinese-born *n* = 602	Australian-born *n* = 602
Mothers	Mean (SD)	Mean (SD)
BMI of mother at start of pregnancy***	21.45 (4.07)	24.9 (5.18)
BMI at survey completion***	23.16 (5.24)	26.00 (5.47)
	Proportion (%)	Proportion (%)
Age (years)***		
15–24 years	2.7	12.0
25–29 years	27.2	24.6
30–34 years	40.2	35.6
35+ years	29.9	27.8
Socioeconomic disadvantage (quintiles) ***		
1st quintile (greatest disadvantage)	13.3	13.8
2nd quintile	7.7	15.9
3rd quintile	26.7	23.3
4th quintile	20.9	21.1
5th quintile (least disadvantage)	31.4	26.0
Main language (English) ***	15.0	100.0
Spouse usually living in house (yes)	94.3	92.2
Spouse at time of birth (yes) *	96.0	93.3
Highest school year completed***		
Year 12 or equivalent	94.4	76.9
Year 11 or equivalent	1.8	10.1
Year 10 or equivalent	3.3	13.0
Did not go to school	0.5	0.0
Educational qualification (yes) ***	89.9	80.7
Highest qualification***		
Postgraduate	43.5	13.0
Bachelor degree	35.3	39.9
Diploma	14.4	13.4
Certificate	6.8	33.7
Income***		
>$156,000	3.7	10.3
$88,400–$155,999	21.8	34.4
$52,000–$88,399	21.9	28.8
$26,000–$51,900	27.8	15.6
<$25,999	24.8	11.0
Parity***		
One	62.6	43.3
Two	31.5	35.2
Three	5.0	14.5
Four or more	0.9	7.0
Currently smoking (yes) ***		
Daily	0.4	9.0
At least weekly	0.0	1.2
Less often	0.1	2.1
Smoked whilst pregnant (yes) ***		
Daily	0.2	6.7
At least weekly	0.2	1.2
Less often	0.0	1.4

*p < 0.05; **p < 0.01; ***p < 0.001

The mean (±SD) age of infants of Chinese-born and Australian-born mothers was similar (6.69 ± 5.04 months and 7.11 ± 5.54 months, respectively; with a range of 2–25 months). Table 2 displays the feeding practices of infants of Chinese-born and Australian-born mothers. Compared to infants of Australian-born mothers, infants of Chinese-born mothers were younger when they first consumed infant formula, water-based drinks, and fruit juice (*p* < 0.01). A higher proportion of infants of Chinese-born mothers were currently receiving breastmilk (66.0% compared to 59.9% of infants of Australian-born mothers, *p* < 0.01). Regarding the age of solid food introduction; both groups reported low compliance with the Australian Infant Feeding Guidelines suggesting solids be introduced at around 6 months of age [22] with more than two-thirds of both groups introducing solids prior to 6 months (67.8% infants of Chinese-born mothers and 75.6% infants of Australian-born mothers, *p* < 0.05). A lower proportion of infants of Chinese-born mothers had been exposed to soft, semi-solid or solid foods; and those infants that had been exposed were older compared to infants of Australian-born mothers (*p* < 0.001). A higher proportion of infants of Chinese-born mothers had ever consumed infant formula, water, toddler milk, soy milk and fruit juice (*p* < 0.01). A lower proportion had ever consumed cow’s milk (*p* < 0.01).

**Table 2:** Feeding practices in infants of Chinese-born and Australian-born mothers living in Australia

	Infants of Chinese-born		Infants of Australian-born	
	n	Mean (SD)	n	Mean (SD)
Age stopped receiving breastmilk (months)	177	4.60 (3.77)	209	4.09 (4.60)
Age when first drank infant formula products (months)	430	1.16 (2.37)	392	1.69 (2.67) **
Age when first drank cow’s milk (months)	57	10.19 (5.90)	94	11.10 (3.33)
Age when first drank soy milk (months)	28	9.36 (6.12)	9	10.3 (5.48)
Age when first drank water-based drinks (months)	85	5.68 (4.83)	82	8.96 (5.63) ***
Age when first drank fruit juice (months)	174	6.37 (4.07)	104	9.92 (5.11) ***
Age when first ate soft, semi-solid, solid foods (months)	270	5.06 (1.30)	324	4.65 (1.14) ***
	n	%	n	%
Currently receiving breastmilk (yes)	577	66.0	569	59.9**
Age stopped breastmilk (months)				
0–6	133	75.1	159	76.1
7–12	37	20.9	35	16.7
> 12	7	4.0	15	7.2
Ever drunk infant formula products (yes)	481	90.4	484	81.4***
Ever drunk water (yes)	477	82.2	479	72.9***
Ever drunk cow’s milk (yes)	475	13.1	478	20.5**
Ever drunk toddler milk (yes)	488	11.7	484	6.0**
Ever drunk soy milk (yes)	474	5.7	477	2.3**
Ever drunk any water-based drinks (yes)	476	18.3	477	17.0
Ever drunk fruit juice (yes)	476	37.6	479	22.8***
Ever eaten soft, semi-solid, solid foods (yes)	487	56.3	484	67.6***
Proportion of infants introduced to soft, semi-solid, solid foods prior to 6 months (yes)	183	67.8%	245	75.6%*
Ever eaten soft, semi-solid, solid foods ≤ 4 months (yes)	107	39.6%	154	47.5%

Water-based drinks: cordial, soft drink, tea (excludes diluted fruit juice and infant formula products). Note, varying of sample size due to age range of infants which were possibly too young to have been exposed to these infant feeding practices yet

*p < 0.05; **p < 0.01; ***p < 0.001

Results of the regression analyses examining associations between ethnicity and age first exposed to the various liquids and solids; and the association between ethnicity and infant feeding practices are presented in Table 3. Regarding the association between ethnicity and age of exposure; in both unadjusted and adjusted models infants of Chinese-born mothers were younger when they first consumed infant formula, fruit juice and older when they first ate solid foods (*p* < 0.05). Infants of Chinese-born mothers were younger when first exposed to water-based drinks, however this was not significant in adjusted models.

**Table 3:** Multiple linear and binary logistic regression models to examine influence of ethnicity on infant feeding practices in Chinese-born and Australian-born mothers

	Model 1 (unadjusted) *n* = 967		Model 2 (adjusted) *n* = 871	
Variable	B coeff (SE)	95% CI	B coeff (SE)	95% CI
Age stopped receiving breastmilk (months)				
Ethnicity (Ref: infants of Australian-born mothers)	0.52 (0.43)	−0.33-1.37	0.67 (0.40)	−0.12-1.47
Age when first drank infant formula products (months)				
Ethnicity (Ref: infants of Australian-born mothers)	−0.53 (0.18)	− 0.87- -0.18**	− 0.92 (0.21)	−1.33- -0.51***
Age when first drank cow’s milk (months)				
Ethnicity (Ref: infants of Australian-born mothers)	− 0.90 (0.75)	−2.39-0.58	0.80 (0.67)	− 0.54-2.13
Age when first drank soy milk (months)				
Ethnicity (Ref: infants of Australian-born mothers)	−0.98 (2.29)	−5.63-3.67	− 0.32 (2.04)	−4.66-4.01
Age when first drank water-based drinks (months)				
Ethnicity (Ref: infants of Australian-born mothers)	−3.28 (0.81)	−4.88- -1.68***	−2.36 (0.84)	− 4.02- -0.71**
Age when first drank fruit juice (months)				
Ethnicity (Ref: infants of Australian-born mothers)	−3.56 (0.56)	−4.65- -2.46***	−1.89(0.54)	−2.94- -0.80***
Age when first ate soft, semi-solid, solid foods (months)				
Ethnicity (Ref: infants of Australian-born mothers)	0.41 (0.10)	0.21–0.61***	0.22 (0.11)	0.01–0.43*
Variable	OR	95% CI	OR	95% CI
Infant currently receiving breastmilk? (yes)	1.30	1.02–1.66*	0.97	0.67–1.41
Infant ever had formula? (yes)	2.16	1.48–3.16***	2.19	1.32–3.62**
Infant ever had water? (yes)	1.72	1.26–2.34***	2.45	1.55–3.87***
Infant ever had cow’s milk? (yes)	0.58	0.41–0.82**	0.37	0.18–0.78**
Infant ever had toddler milk? (yes)	2.07	1.30–3.31**	3.39	1.60–7.18***
Infant ever had soy milk? (yes)	2.56	1.25–5.22**	1.67	0.60–4.61*
Infant ever had any water-based drinks? (yes)	1.09	0.78–1.51	2.48	1.12–5.49*
Infant ever had fruit juice? (yes)	2.05	1.54–2.71***	4.03	2.50–6.51***
Infant ever had solids? (yes)	0.62	0.48–0.80***	0.41	0.25–0.68***
Given water-based drinks ≤ 4 months? (yes)	3.33	1.74–6.34***	7.77	1.96–30.77**
Given fruit juice ≤ 4 months? (yes)	4.07	2.21–7.50***	3.44	1.14–10.38*
Given solids ≤ 4 months? (yes**)**	0.72	0.52–1.00	0.79	0.50–1.26

Water-based drinks: cordial, soft drink, tea (excludes diluted fruit juice and infant formula products)

Model 1: unadjusted. Model 2 was adjusted for maternal age, level of disadvantage, infant age at survey completion, presence of a spouse pre- and post- birth, educational qualifications, income, parity, current smoking status

*p < 0.05; **p < 0.01; ***p < 0.001

In unadjusted models examining the association of ethnicity on feeding practices; infants of Chinese-born mothers were more likely to be currently breastfed; and had ever had formula, water, toddler milk, fruit juice; water-based drinks and fruit juice ≤4 months old compared to infants of Australian-born mothers. In unadjusted models, infants of Chinese-born mothers were less likely to have ever had cow’s milk, solids and solids ≤4 months old compared to infants of Australian-born mothers. Adjusted models revealed the same significant associations between ethnic group and feeding practices *except* the associations for currently breastfed and solids ≤4 months old which attenuated and were no longer significant.

## Discussion

This is the first known study to compare infant feeding practices of Chinese-born mothers with Australian-born mothers and reveals differences in feeding practices between these two groups. Whilst some feeding practices appear to be health promoting in infants of Chinese-born mothers (e.g. a higher proportion of infants were currently being breastfed); a number of feeding practices were potentially obesity promoting (e.g. a younger age of exposure to infant formula, water-based drinks, and fruit juice). The high proportion of infants of Chinese-born mothers currently being breastfed, ever consuming formula and being exposed to formula may reflect a combined feeding approach.

### Breastfeeding

There is limited data on breastfeeding in Chinese-born immigrants in Australia. It is encouraging that the proportion of Chinese-born mothers still breastfeeding at survey completion when infants were 6–7 months old was higher (66%) in this study compared to older data reporting breastfeeding by only 50% of Chinese mothers living in Australia at 3 months [8] and in another study, 56% of mothers at 6 months [20]. There is international evidence of ethnic variation in breastfeeding practices, however primarily in white compared to Hispanic or Black mothers [33]; Indian, Pakistani, Bangladeshi, Black Caribbean, Black African [34] or a combined Asian group [35]. The higher proportion of any breastfeeding amongst Chinese-born mothers in this study may be attributed to their views that breastfeeding is important to their identity as a mother and beliefs of its superior health benefits to both infant and mother [36, 37]. Qualitative interviews with Chinese born mothers in Victoria showed that mothers favoured breastfeeding for its health, emotional and financial benefits [10]. The current study did not examine prevalence of exclusive breastfeeding, but it is clear that a high proportion of mothers born in both China and Australia will not meet the recommendation of breastfeeding for 12 months given that about one-third of both groups were already not currently breastfeeding at 6-7 months old when the survey was completed. This is concerning given the protective effect of breastfeeding on infant weight status [23] and that even combination feeding under 6 months may increase the risk of being overweight/obese later in life [38].

### Formula feeding

In the current study, infants of Chinese-born mothers were younger when they first consumed infant formula, and a higher proportion had ever consumed infant formula compared to infants of Australian-born mothers. Comparison of these findings internationally is difficult as the evidence is still emerging, however these data are consistent with data from North America where the preference for formula (alone or in combination) compared to breastfeeding in women of Chinese descent (born in Hong Kong) is becoming a concern [39]. Previous Australian data is quite dated (1997), showing 50% of mothers bottle feeding, and 16% combination feeding at 3 months; with reported reasons for bottle feeding largely being inadequate milk supply and ease of feeding practice [8, 20]. Concerning misconceptions about bottle feeding have also been reported such as a large proportion of Chinese mothers believing Australian infant formula was better quality compared to that in China, and that a fat baby was a healthy baby [8]. Despite believing that breast milk is best, Chinese mothers recently reported that they thought infant formula was a useful addition in infant feeding as other people could be involved in feeding, babies could sleep through the night and they felt assured of knowing how much the baby has drunk [10]. However, high protein formula feeding has been associated with rapid weight gain and risk of adiposity [25, 27, 28]. Formula feeding (alone or in combination) has been shown to increase risk of overweight and obesity in children [38]. Further research is required to ascertain a deeper understanding of the complex influences behind formula feeding in this Chinese-born group, and whether the infant feeding practices reflect combination feeding of breast and formula.

### Introduction of solids

Overall the average age of soft, semi-solid or solid introduction was younger than recommended in the Australian Infant Feeding Guidelines [22] in both groups of mothers. Recent reviews suggest that introducing complementary foods to infants very early (before 4 months of age) may increase the risk of a child being overweight; however findings are not conclusive [17, 30]. Early introduction of solids may also result in less time breastfeeding, a decline in breastmilk production and sometimes under-nutrition and an increased risk of developing allergies [40]. Compared to infants of Australian-born mothers, a lower proportion of infants of Chinese-born mothers had been exposed to solids; in addition to being older when first exposed. This ethnic diversity in the age of solid introduction has not previously been reported for infants of Chinese immigrants. Qualitative work with Chinese-born mothers in Victoria reported that mothers follow healthcare professional advice to introduce solids between 4-6 months because they want to reduce risks in infants developing allergic diseases [10]. Most commented that prior to 6 months of age, solids were introduced for taste rather than as an important source of nutrients in the infant’s diet [10]. Mothers from all ethnic minority groups (e.g. Indian, Pakistani, Bangladeshi, black Caribbean, black African) have been reported to be less likely to introduce solids before the recommended age than white mothers [41]. Further research on infants of Chinese immigrants, including capturing anthropometric data, is required to understand the risk of early introduction of solids in relation to adiposity in this population.

### Other liquids – Water-based drinks, fruit juice

The Australian Infant Feeding Guidelines do not recommend fruit juice for infants under 12 months of age as it is unnecessary and may interfere with consumption of breastmilk or formula and risk damage to emerging teeth; and it is not recommended to offer tea, herbal tea, coffee, soft drinks, cordial or other beverages [22]. In the current study, a large proportion of mothers, regardless of ethnicity, did not adhere to these guidelines. At the time of survey completion when infants were 6-7 months old, almost one fifth of infants had ever consumed water-based drinks (e.g. cordial, soft drink, tea); a higher proportion of infants of Chinese-born mothers had ever consumed fruit juice. Infants of Chinese-born mothers were younger when exposed to water-based drinks and fruit juice. The present findings support and extend the findings of previous qualitative work with Chinese-born mothers in Victoria which reported that mothers faced pressure from grandparents to introduce water-based drinks to their infants prior to 4 months of age [10].

In the US, one quarter of infants had consumed sugar-sweetened beverages (SSBs) during infancy (between 1-12 months of age) [42]. Examination by ethnicity in the UK has shown some variation in consumption of SSBs - compared to white British counterparts, Pakistani infants had increased consumption of SSBs at 12 months; and increased consumption of SSBs and fruit juice at 18 months [43]. Hispanic children have also been found to consume significantly more juice than non-Hispanic white children [44]. Few studies have examined the association between SSB intake during infancy and obesity during early childhood, however recently SSB consumption during infancy was associated with increased odds of obesity at age 6 years [42]. Numerous studies have also revealed fruit juice consumption in early childhood to be associated with unhealthy weight gain in young children [45–47].

### Strengths and limitations

This study contributes important knowledge regarding differences in infant feeding practices of Chinese-born mothers compared to their non-immigrant counterparts living in Australia. A particular strength of the study is that the data was drawn from a large national database which allows good generalisability; and also permitted adjustment for a large number of covariates. However, we do acknowledge several study limitations. Due to the cross-sectional nature of the database, causality cannot be inferred. Other limitations include recall bias and social desirability bias inherent to self-reported surveys, potential unmeasured confounding factors (e.g. lack of physical activity data collected, year of arrival) and an absence of infant anthropometry data for rate of growth.

## Conclusion

The determinants of child and adult obesity are multifactorial and include modifiable risk and protective factors beginning with infant feeding behaviours [47]. The early infant feeding practices of Chinese-born mothers, the third largest immigrant group in Australia, have not been well documented. The present study found supporting evidence of potential protective practices such as breastfeeding and obesity promoting feeding practices such the consumption of formula, toddler milk, fruit juice and water-based drinks. The contradictory findings of more breastfeeding yet more exposure to formula in infants of Chinese-born mothers is likely to reflect more combination feeding in this ethnic group. The early introduction of fluids other than breast milk but the later introduction of solids highlight both the complexity of feeding norms and the importance in seeking to classify feeding practices accurately in Chinese immigrants living in Australia. This study identifies key modifiable dietary targets for the design of culturally tailored interventions to promote infant health in this immigrant population. Further research is now required to establish a more nuanced understanding of the influence of ethnicity on these infant feeding practices, and their association with risk of adiposity later in life.

## Abbreviations

BMI: Body Mass Index
MCH: Maternal Child Health
SEIFA: Socio-Economic Indexes for Areas
SSB: Sugar-sweetened beverage
